# Clinical evaluation of an endorectal immobilization system for use in prostate hypofractionated Stereotactic Ablative Body Radiotherapy (SABR)

**DOI:** 10.1186/s13014-015-0426-4

**Published:** 2015-05-30

**Authors:** Alexandru Nicolae, Melanie Davidson, Harry Easton, Joelle Helou, Hima Musunuru, Andrew Loblaw, Ananth Ravi

**Affiliations:** Department of Medical Physics, Odette Cancer Centre, Sunnybrook Health Sciences Centre, Toronto, Ontario Canada; Department of Radiation Oncology, Odette Cancer Centre, Sunnybrook Health Sciences Centre, Toronto, Ontario Canada; Department of Radiation Oncology, The University of Toronto, Toronto, Ontario Canada

**Keywords:** SABR, Prostate cancer, SBRT, Immobilization techniques, Rectal retraction

## Abstract

**Background:**

The objective of this study was to evaluate a novel prostate endorectal immobilization system (EIS) for improving the delivery of hypofractionated Stereotactic Ablative Body Radiotherapy (SABR) for prostate cancer.

**Methods:**

Twenty patients (*n* = 20) with low- or intermediate-risk prostate cancer (T1-T2b, Gleason Score < 7, PSA ≤ 20 ng/mL), were treated with an EIS in place using Volumetric Modulated Arc Therapy (VMAT), to a prescription dose of 26 Gy delivered in 2 fractions once per week; the intent of the institutional clinical trial was an attempt to replicate brachytherapy-like dosimetry using SABR. EBT3 radiochromic film embedded within the EIS was used as a quality assurance measure of the delivered dose; additionally, prostate intrafraction motion captured using pre- and post-treatment conebeam computed tomography (CBCT) scans was evaluated. Treatment plans were generated for patients with- and without the EIS to evaluate its effects on target and rectal dosimetry.

**Results:**

None of the observed 3-dimensional prostate displacements were ≥ 3 mm over the elapsed treatment time. A Gamma passing rate of 95.64 ± 4.28 % was observed between planned and delivered dose profiles on EBT3 film analysis in the low-dose region. No statistically significant differences between treatment plans with- and without-EIS were observed for rectal, bladder, clinical target volume (CTV), and PTV contours (*p* = 0.477, 0.484, 0.487, and 0.487, respectively). A mean rectal V80% of 1.07 cc was achieved for plans using the EIS.

**Conclusions:**

The EIS enables the safe delivery of brachytherapy-like SABR plans to the prostate while having minimal impact on treatment planning and rectal dosimetry. Consistent and reproducible immobilization of the prostate is possible throughout the duration of these treatments using such a device.

## Background

There is a growing body of evidence indicating that prostate adenocarcinoma cells exhibit a low α/β ratio suggesting sensitivity to hypofractionated doses of radiation [1–3]. This has driven a recent trend towards higher fractional doses in the form of Stereotactic Ablative Body Radiotherapy (SABR), for localized prostate cancer. A prospective clinical trial was started within our institution prescribing 26 Gy to the prostate in 2 fractions, given one week apart, for patients with localized disease (ClinicalTrials.gov ID: NCT02031328). The intent of this trial was to replicate the dosimetric and rectal tissue-sparing advantages of High Dose Rate (HDR) brachytherapy-like SABR plans. Previous studies have been successful in delivering HDR brachytherapy-like SABR plans using CyberKnife [4]; however, limited access to these specialized devices prevents widespread use of such techniques. Increased access to brachytherapy-like SABR treatments would result from an ability to deliver these treatments using a standard linear accelerator (Linac) [5–9].

In HDR brachytherapy, sources are placed directly within catheters implanted in the prostate, making patient and prostate motion relatively inconsequential to treatment delivery. In SABR however, radiation dose is delivered externally and accurate targeting hinges on adding a safety margin, or planning target volume (PTV), around the prostate to account for movement and setup uncertainties [10]. The expansion of the treatment volume in the vicinity of organs-at-risk (OAR), like the bladder and rectum, may increase the risk of developing unacceptable late urinary and rectal complications, particularly with such large fractional doses [11]. The PTV margin may be confidently reduced if a method of limiting the extent of prostate motion during treatment was consistently possible. One proposed immobilization strategy, and the focus of this study, is to address rectal motion and filling, which has consistently been shown to be a significant predicator of prostate motion [12, 13].

Endorectal balloons (ERB) have been used as a method of controlling the volume and position of the rectum and therefore indirectly immobilizing the prostate [14–16]. While this strategy has generally demonstrated efficacy in multi-fraction treatment regimens there is some debate regarding the reproducibility of positioning [17]; additionally, the process of inflating the ERB raises the anterior surface of the rectum into the high dose region of the treatment plan [18]. This associated deformation of local anatomy makes it more difficult to achieve the tighter dosimetric constraints used in HDR brachytherapy [17–20]. While controversy remains regarding the protective nature of the displaced normal rectal tissue, it is generally agreed that the dosimetric goal is to lower the dose to the rectal volume as this results in lower rates of rectal toxicity [10, 21–24]. An alternative to the ERB has been the use of injectable/implantable spacers or polymeric gels. Although the rectal doses using this system are lower due to the physical separation of the prostate from the rectum, no evidence is available that any substantial prostate immobilizing effect is produced [25, 26]. These methods are also quite invasive, requiring a transperineal incision or interstitial needle placement, and introduce additional departmental costs.

As part of the institutional clinical to replicate brachytherapy-like dosimetry using a standard Linac a prostate endorectal immobilization system (EIS) was developed and evaluated with the intent of immobilizing the prostate gland during each treatment fraction. The system is additionally capable of in-vivo quality assurance (QA) during treatment delivery using radiochromic EBT3 film. The aims of this study were to 1) evaluate the effectiveness of the immobilization device in limiting prostate intrafraction motion over the duration of treatment delivery; 2) to evaluate if plans with similar dosimetric quality can be achieved with- and without the EIS in place; and 3) to evaluate whether accurate, in-vivo dose verification - as an additional dosimetric QA measure– is possible using the EIS.

## Methods

A sample size of 20 patients, with low- or intermediate-risk prostate cancer (T1-T2b, GS < 7, PSA ≤ 20 ng/mL), were treated in a prospective, single-institution clinical trial. Prescription dose was 26 Gy delivered in 2 fractions once a week to the prostate clinical target volume (CTV). Prospective planning was performed on Computed Tomography (CT) data obtained during simulation both with and without the EIS system. CT datasets were obtained without-EIS as a) a precautionary measure in the event that patients were unable to tolerate the EIS, b) stringent planning criteria were not achievable with the EIS in place, and c) for dosimetric comparison to EIS treatment plans. Patients excluded from the trial, due to the previously stated criteria, were offered an institution standard 35 Gy in 5 fraction SABR treatment as an alternative. Retrospective data analysis of prostate displacements that occurred during treatment was performed using Conebeam Computed Tomography (CBCT).

### Simulation & planning details

Three gold fiducial markers were implanted transperineally under Transrectal Ultrasound (TRUS) guidance prior to CT simulation to act as surrogates for prostate position and to aid in daily image guidance. Patients were instructed to have a comfortably full bladder and also given a self-administered enema and simethicone prior to the simulation process. CT scans were acquired with the patient in a supine position with their legs supported in a custom-made Vac-lok mounted on a blue Kneefix 2 (CIVCO Medical Soln., Coralville, IA, USA).

### EIS immobilization system

**Fig. 1:**
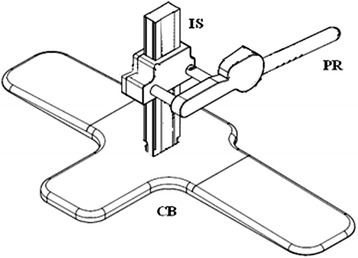
Endorectal Immobilization System. 3D Mechanical drawing of the EIS system highlighting the three main components of the system: The carbon-fibre base (CB), the indexed mechanical system (IS), and the probe apparatus (PR)

The EIS system (Fig. 1) was mounted on the CT simulator couch using a rigid carbon-fibre base. A mechanical indexing system enabled the 5-degree-of-freedom translation and angulation of the probe. The probe was composed of a USP class VI medical-grade photopolymer: Accura® Clearvue™ (3D Systems, Inc., South Carolina, USA). The radiographic properties of this material have been previously validated by our group [27]. The probe external diameter was of a similar size to a Transrectal Ultrasound (TRUS) probe commonly used during HDR brachytherapy image guidance – 20 mm (Transducer Type 8848, Analogic Corporation, Boston, MA, USA) - ensuring a consistent rectal diameter. Four small tungsten beads were embedded within the probe to aid in CBCT localization at the time of treatment; a slot for GafChromic EBT3 film (Covington, KY, USA) was also available. The probe assembly was inserted with the patient lying either supine or decubitus and then affixed to the indexing system, and subsequently the base. A second CT scan was then acquired without the EIS system in place. The tip of the probe assembly was inserted to approximately mid-gland before being affixed to the indexing system. In an attempt to counteract the slight volume deformations produced with an intraluminal device, purposeful distension of the rectum away from the high-dose regions posterior to the prostate were applied with the intent of improving rectal dosimetry. Typical distensions achieved were on the order of 2–3 cm in the posterior direction with angulations ranging from 10-25° posteriorly.

Pinnacle3 (version 9.2, Koninklijke Philips NV, Eindhoven, Netherlands) was used for treatment planning. Prostate contours were segmented on the CT simulation data set; the clinical target volume (CTV) was equivalent to the prostate contour and did not include proximal seminal vesicles. A 3 mm isotropic expansion of the CTV was used to generate a PTV margin. The selection of a 3 mm isotropic PTV margin was primarily due to the reduction in intrafraction prostate motion observed when transitioning from an IMRT to a VMAT delivery method; a center-specific 5 mm margin for SABR patients receiving 35 Gy-in-5 fractions was therefore reduced to 3 mm to aid in minimizing normal tissue toxicities [28]. In addition the three gold fiducial markers were contoured as well as OARs. OARs of interest included the bladder, rectum, and small bowel. The rectal volume was delineated by contouring the entire rectum from the bottom of the ischium to the sigmoid flexure (a length of approximately 11–12 cm); the volume within the EIS probe apparatus, was subtracted from the rectum contour to analyze only the rectum wall and contents and not the probe material. Without the aid of magnetic resonance (MR) imaging contouring the entire volume offered a conservative estimate of the rectal membrane position for plan optimization as opposed to approximating a rectal membrane. Both sets of plans used “Smart Arc” optimization with the goal of maintaining equal target coverage while minimizing rectal dose parameters. Volumetric Modulated Arc Therapy (VMAT) was used to treat the CTV to a prescription dose of 26 Gy in 2 fractions (assuming an α/β ratio of 1.4 [2]), giving an EQD2 (Equivalent dose when given in 2 Gy fractions) of 110 Gy [29, 30].

### Treatment delivery

Patients were treated on an Elekta Beam Modulator with a HexaPOD-enabled couch capable of roll, pitch, and yaw corrections (Elekta AB, Stockholm, Sweden). Cone Beam Computed Tomography scans were acquired directly before and after the treatments to evaluate prostate intrafraction motion. CBCT datasets were first co-registered to the EIS to verify position and depth; disagreements between positioning at treatment and that at planning required fine manual adjustments to the probe depth to bring the setup in accordance with the desired planning position prior to treatment delivery. CBCT datasets were then re-acquired and co-registered to the three fiducial markers using a grey-value registration algorithm for coarse positional adjustment and manual positioning reserved for fine adjustment, followed by visual examination of the prostate-rectum interface.

### Intrafraction prostate displacement

Intrafraction prostate displacements were computed for each patient by taking the difference between translations and rotations obtained from pre-treatment CBCT and post-treatment CBCT co-registrations, respectively [31]. Descriptive statistics (mean, standard deviation, 95 % confidence interval (CI), and outlying data points), were computed for each patient in the Left/Right (L/R), Superior/Inferior (S/I), and Anterior/Posterior (A/P) translational axes. The magnitude of the intrafraction motion was computed by summing the displacements in each translational axis in quadrature. The magnitude of the motion was plotted as inverse-cumulative frequency histograms to determine the range of intrafraction motion for all patients. In total 40 CBCT pre-post scan combinations were examined.

### Dosimetric analysis with- and without-EIS

A subset of patients (*n* = 10) were planned with and without the EIS with the intent of evaluating its effects on dosimetry and plan quality. Plans were completed using the same prescription dose with the intent of maintaining similar target coverage; the impact on OAR dose, with emphasis on the rectum, was then examined. Prostate CTV and PTV coverage were examined using the conformity index and rectal and bladder V80% in cubic centimeters (cc) [7, 10]. Conformity indices were calculated using the V100% and V85% divided by the CTV and PTV volumes, respectively. The average Dose-Volume Histogram (DVH) for each OAR and target with and without the EIS was generated using the Computational Environment in Radiation Therapy Research (CERR, ©2013, Deasy J) software. A one-tailed Student’s *t*-test (significance level α = 0.05), was used to evaluate differences between average DVH curves for the CTV, PTV, rectum, and bladder for patients treated with- and without the EIS.

### In-vivo rectal dosimetry

Using radiochromic film for patient specific quality assurance is a proven method for ensuring the quality of delivered plans by measuring absolute dosimetry [32–34]. Films were evaluated after delivery of every fraction, as a quality assurance measure of the delivered dose. 20, 150 mm × 38 mm GafChromic EBT3 film strips were evaluated using FilmQA software (Ashland, Covington, KY, USA). The irradiated film was compared to the dose grid (generated from Pinnacle 3), taken in the plane of the measured film. The in-plane dose grid was calculated by software developed in-house. A Gamma index passing rate with thresholds of 3 % and 3 mm was used in this study to evaluate the agreement between the predicted dose computed with Pinnacle3 and the actual delivered dose [35].

## Results

### Intra-fraction prostate displacement

**Fig. 2:**
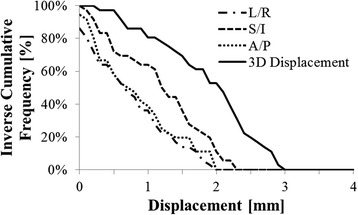
Intrafraction prostate displacement with EIS. Inverse Cumulative histogram of the 3D displacement, and left-right (L/R), superior-inferior (S/I), and anterior-posterior (A/P) axis displacements. Less than 1 % of 3D displacements were greater than 2.9 mm, and no displacements were ≥ 3.0 mm. Similarly, no translational displacements were ≥ 2.0 mm in the L/R and A/P axes, and none were ≥ 2.3 mm in the S/I axis

Intrafraction motion values were computed as mean ± one standard deviation (SD), followed by the 95 % confidence interval (CI). Negative values corresponded to shifts in the right, inferior, and anterior directions. Intrafraction translational displacements were −0.20 ± 0.97 mm (CI = ± 1.89 mm) in the L/R axis, −0.08 ± 1.40 mm (CI = ± 2.69 mm) in the S/I axis, −0.28 ± 1.04 mm (CI = ± 2.04 mm) in the A/P axis. The mean 3D displacement magnitude was 1.83 ± 0.75 mm (CI = ±1.47 mm). Less than 5 % of 3D displacements were ≥ 2.87 mm, less than 1 % were ≥ 2.90 mm and no displacements were ≥ 3.00 mm. Similarly, in the L/R and A/P axes no displacements were ≥2.00 mm and no displacements in the S/I axis were ≥ 2.30 mm. These results are summarized in Fig. 2.

### With- and without-EIS dosimetric analysis

**Fig. 3:**
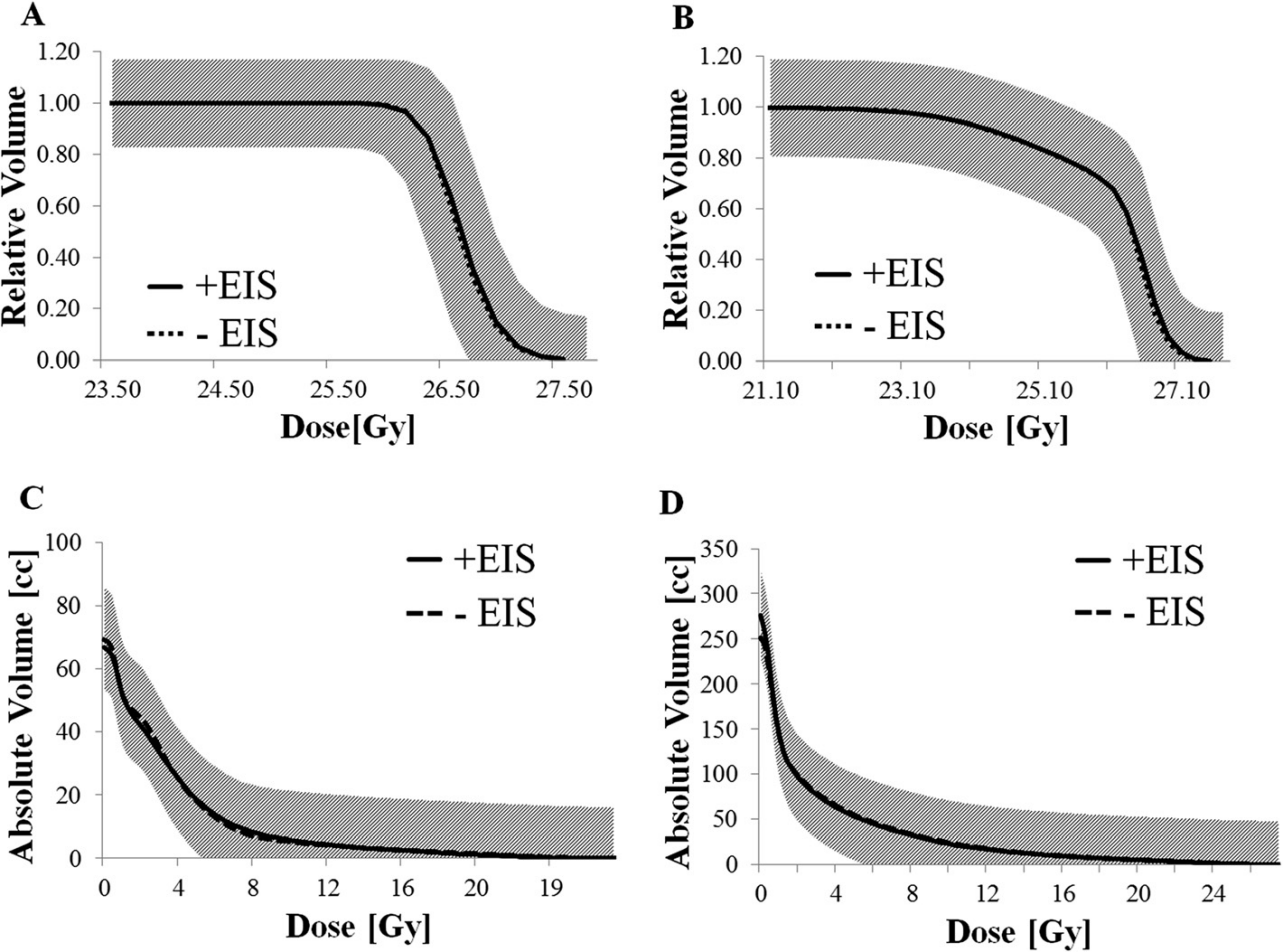
Target and OAR doses with- and without-EIS. **a & b** Comparison of average Dose-Volume Histogram (DVH) results for 10 patients planned with- and without EIS (dashed lines). Prescription is 26 Gy ≥ 99 % to the clinical target volume (CTV) and 22 Gy ≥ 99 % to the planning target volume (PTV), respectively. Average DVH results for the Rectal (**c**), and Bladder (**d**), contour with- and without EIS are also presented. Standard deviation bands (1SD) are displayed as grey areas (without EIS), and hatched areas (with EIS). Overlapping standard deviation bands are displayed as grey

**Fig. 4:**
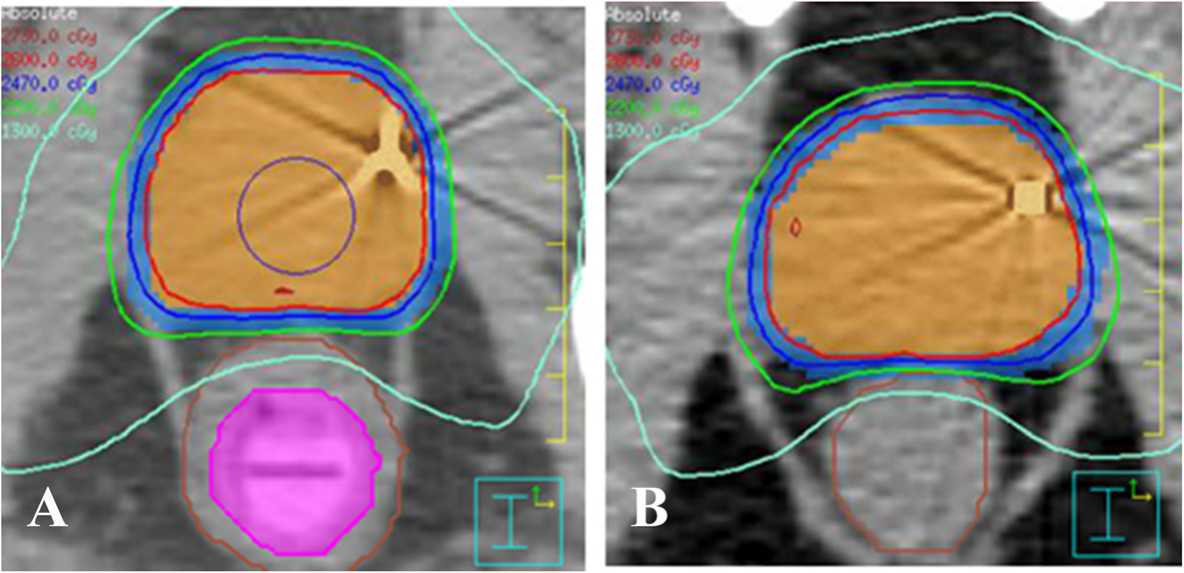
Sample treatment plans of patients treated with- and without-EIS. **a** Treatment plan showing isodose distribution for patient treated with the EIS (purple). Displayed are the prostate CTV (orange), and PTV (blue), as well as the overall rectal contour. Percent isodose lines displayed are 105 % (maroon), 100 % (red), 95 %(dark blue), 85 % (green), and 50 % (light blue). **b** Isodose distribution for a patient treated without the EIS. CTV prescription dose is 2600 cGy

Figure 3(a & b) presents the average DVH results with- and without the EIS for the prostate contour (CTV and PTV) for 10 patient plan sets. Figure 3(c) presents the average DVH results for the rectum contour in absolute volume (cubic centimeters), and Fig. 3(d) presents the average DVH results with and without the EIS for the bladder contour. No statistical differences were evident between the dosimetry with and without the EIS for the bladder, rectum, CTV and PTV (*p* = 0.477, 0.484, 0.487, and 0.487, respectively). Similarly, the average CTV conformity index was 1.11 ± 0.03 for both groups; the average PTV conformity index was 1.13 ± 0.03 and 1.12 ± 0.03 for the groups with- and without- EIS, respectively. A transverse representation of the calculated dose distributions near the prostate midplane for both sets of plans can be found in Fig. 4.

### In-vivo film analysis

**Fig. 5:**
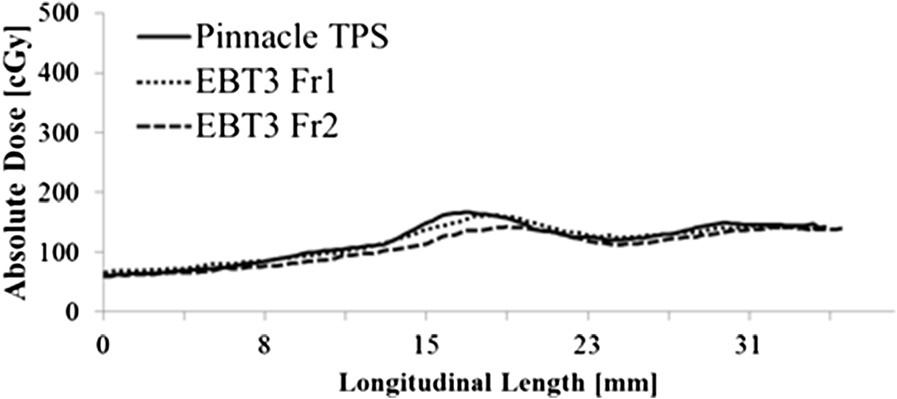
Longitudinal dose profile for EBT3 film sample. Absolute dose for a single patient as a function of the longitudinal film length plotted against the computed dose model. Good agreement was seen between EBT3 and the treatment planning system (TPS) model for the first and second fractions (Fr). Slight differences in curves were likely due to minute changes in EIS positioning from that at planning

Evaluation of in-vivo, irradiated EBT3 films demonstrated an average gamma passing rate of 95.64 ± 4.28 %. Figure 5 shows a sample dose-profile for a single patient comparing our predicted planar dose model (derived from Pinnacle 3), on the longitudinal film axis to the same axis on the irradiated film. Despite the inevitable small deviations in probe - and therefore EBT3 film plane - alignment from that at planning comparable dose profiles were obtained in the region of high-dose gradient posterior to the treated volume.

## Discussion

A comparison of prostate motion observed between patients treated using the EIS and those using the ERB, one of the most common prostate immobilization methods, is unavoidable. A similar mono-institutional study conducted at our centre evaluated 30 intermediate-risk prostate cancer patients treated with a single SABR boost, using VMAT, in dose-escalation (10 Gy, 12.5 Gy, and 15Gy) [36]. In that study an ERB was used for prostate immobilization during the treatment and CBCT image guidance utilized to measure intrafraction prostate motion. This trial showed 3D prostate displacements of 2.61 ± 1.50 mm (CI = ± 3.10 mm) [36]. In comparison, 3D prostate displacements observed in patients treated using the EIS were significantly smaller at 1.83 ± 0.75 mm (CI = ± 1.47 mm), following a two-tailed Student’s *t*-test (p = 0.023, significance level α = 0.05). The fractional dose, and therefore treatment time was comparable for both studies: 13 Gy per fraction compared to patients stratified into doses ranging from 10–15 Gy per fraction, both at > 6-min treatment times. None of the treated fractions had 3D displacements ≥ 3 mm in the current study. These results are comparable with a number of publications using endorectal balloons for prostate immobilization, summarized in Table 1. In essence, this method offered robust and consistent intrafraction immobilization, highly comparable to that of the ERB, and with minimal changes as a function of increased treatment times. It is hypothesized that the underlying mechanism behind this immobilization was the direct fixation of the rectum to the treatment couch, which additionally limited any involuntary pelvic movement by patients. One of the limitations of the intrafraction motion study was the use of pre- and post-CBCT methods for evaluation. Because prostate gland motion may not be uniform or linear over the treatment duration, and in fact may be somewhat random in nature, the net displacement of the prostate over the treatment duration may potentially be underestimated using a pre-post imaging design [37]. Cine-MRI, now available at our center, has been shown to be an effective method for live-tracking of prostate intrafraction motion [38]. A valuable follow-up study would involve using cine-MRI to verify the displacement results obtained in this study by tracking the prostate motion over the elapsed treatment time, thereby eliminating the temporal limitations imposed by the pre-post CBCT-determined motion.

**Table 1:** Comparing prostate intrafraction motion magnitude. Prostate intrafraction motion results from multiple institutional trials using an endorectal balloon (ERB) for immobilization compared to the current study using an EIS. Patients treated with the EIS showed significantly reduced 3D intrafraction motion for treatment times longer than 6 min. EM = Electromagnetic

Study	Sample size (n)	Technique	Immobilization	Motion Evaluation	3D intrafraction motion	Treatment time (min)
Current study	20	VMAT	EIS	Pre-post CBCT	0 % > 3 mm	>6
Chiang et al. 2014 [36]	30	VMAT	ERB	Pre-post CBCT	3.7 % > 4.5 mm	>6
Smeenk et al. 2012 [15]	30	IMRT	ERB	Real-time EM tracking	7.0 % > 3 mm	<6
Wang et al. 2012 [34]	30	IMRT or VMAT	ERB	Real-time EM tracking	5.0 % > 3 mm	≤6
Both et al. 2011 [14]	24	IMRT or VMAT	ERB	Real-time EM tracking	5.2 % > 3 mm	<6

Similar studies reporting on the use of rectal retraction as a means of limiting rectal dose demonstrated improved dosimetry [39, 40]; however, in the present study the differences between clinically relevant CTV, PTV, rectum and bladder dosimetric parameters with and without the EIS system were minimal (Fig. 3). Absolute volume of irradiated tissue was evaluated to provide a point-of-comparison to HDR brachytherapy and has also been found to better approximate the extent of rectal toxicities, which are volume-dependent [11]. As noted previously the diameter of the EIS probe was similar to the 20 mm diameter TRUS probes used in ultrasound-guided HDR brachytherapy procedures, this similarity ensured that average rectal volumes as well as distension were roughly identical. With similar average rectal volumes during SABR (with the EIS in place), and HDR brachytherapy (with a TRUS in place), the primary contributor to the amount of rectal tissue exposed to high doses of radiation was the positioning of the rectum. Posterior retraction away from the high-dose region is applied during treatment with an EIS in order to minimize rectal dose. During HDR brachytherapy compression of the rectum towards the high-dose region is required in order to obtain adequate image quality, this should theoretically increase the dose to the rectum. However, as a result of the rapid dose fall-off in HDR brachytherapy the volumes of rectal tissue within the high dose region were comparable between both treatment modalities. The mean rectal volumes obtained by excluding the EIS lumen from the rectal volume analysis (66.7 cc vs. 69.4 cc for plans with and without EIS, respectively) were comparable. The rectal V80% was lower for plans with the EIS with a mean value of 1.07 cc, and 1.27 cc for plans without the EIS; however, these differences were statistically insignificant. The rectal V80% values are comparable to similar HDR brachytherapy regimens, which typically aim to achieve a rectal V80% < 1 cc [7]. In order for the SABR-with-EIS treatment plans to approach a rectal V80% typically achieved using HDR brachytherapy an advantageous retraction away from the high-dose region is required.

With use of an ERB there is an associated increase in dose delivered to the anterior rectal mucosa; this was not evident with the use of the EIS system as the volume of anterior rectal mucosa receiving the higher doses was reduced by distending the rectum away from the prostate. This has the added benefit of simultaneously increasing the distance of the posterior rectal mucosa from the high-dose region, potentially further reducing the overall rectal dose. Future evaluations of the EIS system will use MR imaging to define rectal contours with improved accuracy and provide better estimates of the impact on rectal dosimetry, potentially providing more concrete evidence of this phenomenon. From this study it was observed that target immobilization can be achieved using the EIS system without compromising target and OAR dosimetry; thereby, allowing the delivery of ablative treatments with brachytherapy-like rectal tissue sparing.

The delivery of large doses per fraction of radiation without a number of quality assurance measures in place is potentially problematic: accurate delivery of dose to the target and avoidance of critical structures is typically evaluated during follow-up by patient-reported side-effects scores. To provide a more objective in-vivo dose monitor during treatment a QA measure was directly incorporated into the EIS system to ensure that the calculated treatment plans were being accurately delivered. This was achieved using EBT3 radiochromic film measurements. High dose resolution (up to about 10 Gy) is possible using EBT3 films and was therefore well-suited to measuring the rapid dose gradient of the SABR distribution [31–34]. Despite the small probe positioning deviations previously discussed the vast majority of films showed good agreement with the Pinnacle3-determined planar dose (average gamma passing rate > 95.64 ± 4.28 %). These results are comparable to generalized Intensity Modulated Radiation Therapy (IMRT) plans evaluated in phantoms using similar quality metrics [41]. Figure 5 shows an example of a typical dose profile obtained during treatment relative to the computed planar dose in Pinnacle3, good spatial agreement in the longitudinal central-axis of the film was evident. Measurement of the low doses received by the film with known spatial orientation relative to the high-dose treatment volume was relatively robust to minor variations in probe positioning.

## Conclusions

The novel EIS system provided immobilization of the prostate gland highly comparable to the immobilization provided by an ERB when used in hypofractionated SABR of the prostate. The system does not adversely impact target and OAR dosimetry, thereby allowing a means of sparing of rectal tissue while simultaneously delivering high fractional doses of radiation. Future evaluations using MR imaging for more accurate delineation of rectal wall tissue will provide a clearer picture of the dosimetric impacts of the EIS system in the high-dose regions. The adoption of this system has the potential to greatly improve the accuracy of prostate SABR delivery for patients with localized prostate cancer.

## Abbreviations

EIS: Endorectal immobilization system
SABR: Stereotactic ablative body radiotherapy
HDR: High-dose-rate
Linac: Linear accelerator
PTV: Planning target volume
ERB: Endorectal balloon
QA: Quality assurance
GS: Gleason score
PSA: Prostate specific antigen
CTV: Clinical target volume
CT: Computed tomography
CBCT: Conebeam computed tomography
TRUS: Transrectal ultrasound
OAR: Organ-at-risk
VMAT: Volumetric modulated arc therapy
L/R: Left/right
S/I: Superior/inferior
A/P: Anterior/posterior
DVH: Dose-volume histogram
SD: Standard deviation
CI: Confidence interval
Cc: Cubic centimeters
IMRT: Intensity modulated radiation therapy
